# Emergence and Evolution of Triple Reassortant Highly Pathogenic Avian Influenza A(H5N1) Virus, Argentina, 2025

**DOI:** 10.3390/v18050525

**Published:** 2026-04-30

**Authors:** Estefania Benedetti, Maria Carolina Artuso, Alex Byrne, Maria de Belen Garibotto, Martín Avaro, Luana Piccini, Ariana Chamorro, Marcelo Sciorra, Vanina Marchione, Mara Russo, Maria Elena Dattero, Erika Macias Machicado, Monica Galiano, Nicola Lewis, Andrea Pontoriero

**Affiliations:** 1 Instituto Nacional de Enfermedades Infecciosas “Dr. Carlos G.Malbrán”, Servicio de Virosis Respiratorias, Departamento de Virología, Buenos Aires C1282AFF, Argentina; mavaro@anlis.gob.ar (M.A.); achamorro@anlis.gob.ar (A.C.); vmarchione@anlis.gob.ar (V.M.); mrusso@anlis.gob.ar (M.R.); medattero@anlis.gob.ar (M.E.D.); emacias@anlis.gob.ar (E.M.M.); aponto@anlis.gob.ar (A.P.); 2 Servicio Nacional de Sanidad y Calidad Agroalimentaria, Dirección General de Laboratorios y Control Técnico, Buenos Aires C1063ACW, Argentina; mcartuso@senasa.go.ar (M.C.A.); mdbg@senasa.gob.ar (M.d.B.G.); lpiccini@senasa.gob.ar (L.P.); msciorra@senasa.gob.ar (M.S.); 3 WHO Collaborating Centre for Reference and Research on Influenza, Crick Worldwide Influenza Centre, The Francis Crick Institute, London NW1 1AT, UK; alex.byrne@crick.ac.uk (A.B.); monica.galiano@crick.ac.uk (M.G.); nicola.lewis@crick.ac.uk (N.L.)

**Keywords:** Argentina, triple reassortant virus, highly pathogenic avian influenza (HPAI), H5N1, one health, molecular evolution

## Abstract

The H5N1 subtype of highly pathogenic avian influenza (HPAI) poses a major zoonotic threat due to its high fatality rate and capacity for cross species transmission. In early 2025, Argentina detected a novel triple reassortant A(H5N1) virus in Chaco Province, combining Eurasian, North American, and South American lineage segments. Genomic analyses of subsequent outbreaks in Buenos Aires and Entre Ríos confirmed persistence of this reassortant and additional HA substitutions (T204K, P251S) potentially linked to increased mammalian receptor affinity. Although PB2 sequences lacked canonical mammalian-adaptive markers (E627K, Q591K, D701N), all contained I292M, a mutation associated with human adaptation. Phylogenetic analyses revealed distinct genotypes and increasing divergence. These findings indicate ongoing viral evolution and adaptation within Argentina, emphasizing the urgent need for sustained genomic surveillance, timely data sharing, and integrated One Health strategies to mitigate zoonotic and socioeconomic risks associated with H5N1 spread in South America.

## 1. Introduction

Avian influenza and the H5N1 subtype in particular, poses a significant public health risk due to its zoonotic potential and the high fatality rate among reported human cases. There is also recent evidence of sustained mammal-to-mammal transmission of highly pathogenic avian influenza (HPAI) [1], which increases the risk of the virus acquiring mutations enabling sustained spread among humans; raising concerns for a potential pandemic [2,3]. Moreover, outbreaks in poultry result in substantial economic losses and represent a critical threat to global food security, emphasizing the necessity of sustained active surveillance, rigorous biosecurity measures, and comprehensive preparedness strategies for potential public health emergencies [4].

Since its first detection in South America in late 2022, clade 2.3.4.4b HPAI H5N1 has caused extensive outbreaks in wild birds and poultry across the continent. In Argentina during 2023, several outbreaks of A(H5N1) clade 2.3.4.4b were reported in birds—including wild birds, backyard poultry, and commercial flocks—as well as in marine mammals (South American sea lions) [5].

In early 2025, Argentina reported the first evidence of a novel triple reassortant A(H5N1) virus in the Chaco Province (located in the northeast of the country), with gene segments derived from Eurasian H5N1 (HA, NA and MP), North American low pathogenic avian influenza (LPAI) (NP) and South American LPAI lineages (PA, PB1, PB2 and NS). Such events highlight the dynamic evolution of H5N1 in the Americas [6].

**Figure 1:**
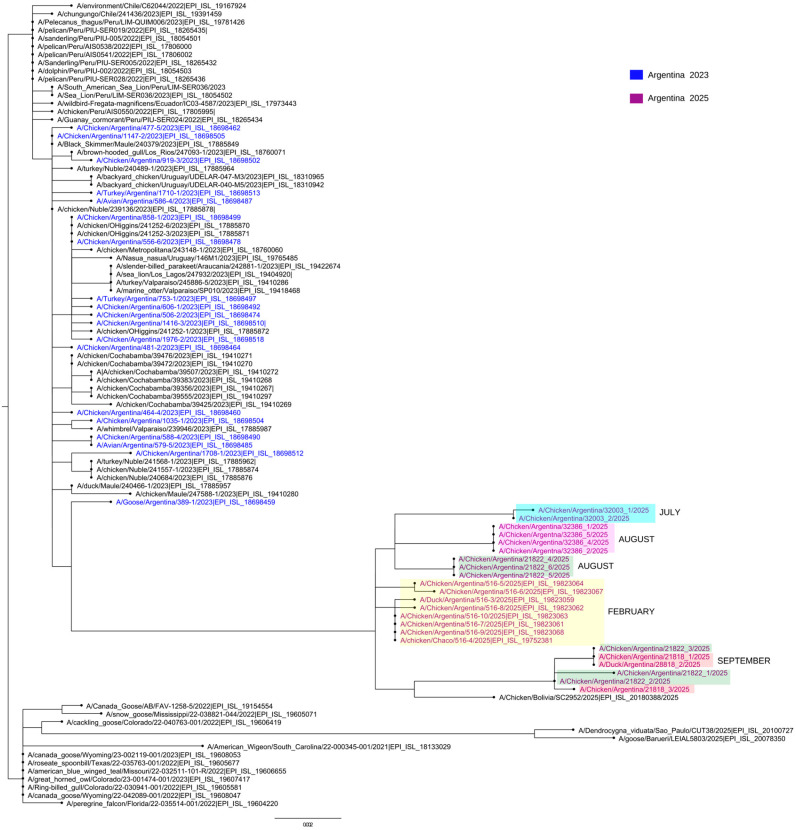
Maximum-likelihood phylogeny of hemagglutinin (HA) gene sequences of HPAI H5N1 viruses detected in Argentina, 2023–2025. Strains from 2023 are shown in blue, while those from 2025 are shown in violet. The months of the 2025 outbreaks are also indicated. Viruses from 2025 form a separate cluster relative to those detected in 2023. The different colors indicate distinct 2025 outbreaks.

In this study, we present a preliminary genomic analysis of H5N1 viruses detected during subsequent outbreaks in Argentina in 2025 (Figure 1). Between July and September, five additional outbreaks were reported in Buenos Aires and Entre Ríos provinces, in the central region of the country, where the triple-reassortant H5N1 virus was also identified. Importantly, the most recent sequences obtained maintained the reassortant constellation while also showing additional amino acid substitutions, highlighting the continued evolution of H5N1 in South America following its introduction into the region.

## 2. Materials and Methods

Tracheal and/or cloacal swabs were collected from domestic birds during official investigations of four avian influenza outbreaks that occurred in Buenos Aires and Entre Ríos provinces between July and September 2025 as part of the active and passive surveillance activities conducted by SENASA. Samples were submitted to the SENASA Official Laboratory for influenza A detection and H5 subtype confirmation by RT-qPCR following the recommendations of the WOAH Manual of Diagnostic Tests and Vaccines for Terrestrial Animals (2022) and the laboratory’s standard operating procedures.

A total of 23 A(H5N1)-positive samples (22 poultry and 1 duck) were selected for whole-genome sequencing. Viral RNA was extracted from clinical material, reverse-transcribed, and amplified using a multisegment RT-PCR approach targeting all eight influenza genome segments. Sequencing libraries were prepared from the resulting amplicons and sequenced on Illumina platforms. Consensus genomes were assembled using IRMA (v1.0.3) with default parameters.

To assess potential adaptive changes, amino acid substitutions in HA and PB2 were screened for known host-specificity markers and other relevant mutations using the GISAID FluServer tool [7]. In addition, all genomes were analyzed with GenoFLU [8] to evaluate genomic constellations and determine whether sequences corresponded to previously recognized H5N1 genotypes or represented novel reassortant combinations.

## 3. Results

A phylogenetic tree of HA sequences including viruses from the July–September 2025 outbreaks and the triple-reassortant strain identified in January 2025 in Chaco Province showed that all recent viruses cluster within the same reassortant lineage but with clear diversification, consistent with ongoing local evolution (Figure 1).

**Figure 2:**
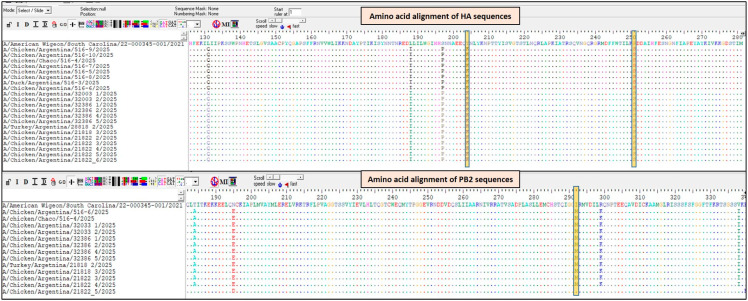
Amino acid alignments of hemagglutinin (HA) and polymerase basic protein 2 (PB2) genes of highly pathogenic avian influenza A(H5N1) viruses detected in Argentina, 2025, compared with reference sequences A/American_Wigeon/South_Carolina/22-000345-001/2021.

Analysis of the HA gene sequences revealed several amino-acid substitutions (Figure 2). The T204K substitution (H5 numbering; position 192 in H3 numbering) is located within the receptor-binding site (RBS), specifically in the 190-helix, a region previously implicated in receptor recognition [9,10]. In contrast, the P251S substitution (H5 numbering; position 239 in H3 numbering) is positioned outside the RBS region.

Regarding the PB2 gene, none of the 12 viruses contained the E627K, Q591K or D701N substitutions, which are well-recognized determinants of enhanced polymerase activity in mammalian hosts [1,11,12]. However, all viruses shared several substitutions, including I292M, which has been identified by the FluServer tool as a host-associated marker (Figure 2).

GenoFLU analysis showed that the 2025 Argentine viruses could not be assigned to any previously described genotype, supporting the occurrence of reassortment events. Considerable genetic divergence was also observed among the sequences.

## 4. Discussion

The genomic characterization of A(H5N1) viruses detected during poultry outbreaks in central Argentina in 2025 demonstrates continued circulation and diversification of the triple-reassortant lineage first reported earlier that year. The presence of the T204K substitution within the 190-helix of the receptor-binding site is notable, as mutations in this region have been shown to modulate receptor-binding properties and influence host specificity [9,10]. However, this substitution alone is not sufficient to confer a human-type receptor-binding phenotype and likely requires the presence of additional changes within the receptor-binding site to significantly affect transmission dynamics. By contrast, the P251S substitution is located outside the receptor-binding site and has not been directly associated with changes in receptor specificity, although potential effects on HA stability or antigenic properties cannot be excluded.

The persistence of this lineage across multiple outbreaks, together with the detection of recurrent HA substitutions in regions implicated in receptor binding, suggests ongoing adaptive evolution. Although classical PB2 mammalian-adaptation markers were absent, all viruses consistently carried the I292M substitution, highlighting the need to further investigate its potential functional significance in the context of clade 2.3.4.4b viruses. This mutation is not considered a canonical mammalian-adaptation marker and has been only sporadically reported in avian influenza A viruses, including some H5Nx lineages.

The inability to classify these viruses into existing genotypes through GenoFLU, along with the observed genomic heterogeneity, reinforces the dynamic nature of H5N1 evolution in the region. This limitation likely reflects novel reassortment patterns and increased genomic divergence, as well as the limited representation of recent South American H5N1 genomes in current reference databases, underscoring both the ongoing evolution of clade 2.3.4.4b viruses.

These findings highlight the importance of sustained genomic surveillance and rapid data sharing to detect emerging variants and assess their potential zoonotic risk. The continued circulation of a triple-reassortant H5N1 clade 2.3.4.4b virus emphasizes the need for strengthened cross-sectoral collaboration between human, animal, and environmental health systems. Ongoing multisectoral coordination within the national surveillance system remains essential to ensure early outbreak detection, risk assessment, and prompt implementation of control measures.

## Data Availability

The sequences generated in this study will be deposited in the GISAID database and will be available.

## Abbreviations

The following abbreviations are used in this manuscript:

HPAI	Highly Pathogenic Avian Influenza
LPAI	Low Pathogenic Avian Influenza
RBS	Receptor Binding Site
